# Intraperitoneal delivery of cannabidiol (CBD) and Δ^9^-tetrahydocannabinol (THC) promotes papillomavirus infections in athymic nude mice

**DOI:** 10.1016/j.tvr.2024.200307

**Published:** 2024-12-16

**Authors:** Sarah A. Brendle, Jingwei Li, Dongxiao Sun, Junjia Zhu, Angela N. Henderson-Redmond, Daniel J. Morgan, Karla K. Balogh, Danielle Covington, Debra A. Shearer, Jiafen Hu

**Affiliations:** a The Jake Gittlen Laboratories for Cancer Research, Pennsylvania State University College of Medicine, Hershey, PA, 17033, USA; b Department of Pathology and Laboratory Medicine, Pennsylvania State University College of Medicine, Hershey, PA, 17033, USA; c Department of Pharmacology, Mass Spectrometry Core Facilities, Pennsylvania State University College of Medicine, Hershey, PA, 17033, USA; d Department of Public Health Sciences, Pennsylvania State University College of Medicine, Hershey, PA, 17033, USA; e Department of Biomedical Sciences, Marshall University, Huntington, WV, 25755, USA; f Department of Comparative Medicine, Pennsylvania State University College of Medicine, Hershey, PA, 17033, USA

**Keywords:** Marijuana, Cannabis, Δ^9^-tetrahydocannabinol (THC), Cannabidiol (CBD), Papillomavirus, Mouse model, Secondary infection, Athymic nude mice, Oral infection, Myeloid cells, Dermal dendritic cells (CD205+), Granulocytes (Ly6G+), And macrophages (F4-80+)

## Abstract

We used our mouse papillomavirus (MmuPV1) model to test the hypothesis that two primary psychoactive ingredients of marijuana, Δ^9^-tetrahydrocannabinol (THC) and cannabidiol (CBD), promote papillomavirus persistence in the oral mucosa of infected mice. We conducted intraperitoneal (ip) injections of a moderate dose (3 mg/kg) of either CBD and/or THC in both male and female athymic nude mice and followed the mice up to 20 weeks post-infection. These doses are comparable to what is estimated for human conventional cannabis consumption. All mice were infected with MmuPV1 in the oral cavity at week 4 post-ip delivery of CBD, THC, or a combination of THC and CBD (T + C). THC and CBD were detected in the blood of treated mice for up to 72 h after ip injection. Significantly higher levels of viral DNA were detected in males from both CBD and T + C-treated groups compared to those in the control group at 9- 10-and 12-weeks post infection. A marginally increased viral RNA was also detected in the infected tongues of males in all tested groups compared to that in males in the vehicle control group; the opposite was observed in females. We detected significantly higher levels of dermal dendritic cells (CD205^+^CD11c^+^), granulocytes (Ly6G^+^), but macrophages (F4-80+) recruited to the infected tongues of CBD-treated females. Our findings suggest that CBD may play a role in promoting MmuPV1 persistence in the oral cavity.

## 1 Introduction

HPV-associated oropharyngeal squamous cell carcinoma (OPSCC) has become endemic in developed countries over the past two decades, despite the significant decline in other subsets of head and neck cancers due to advances in cancer treatments [[1], [2], [3], [4]]. Although HPV infection is not sex-specific, HPV-associated oropharyngeal cancer cases occur predominately in white males. The overall prevalence of oral HPV infection is higher in men (11.5 %) than in women (3.2 %), equating to 11 million men and 3.2 million women nationwide [5], resulting in a higher incidence of OPSCC in men than in women [6]. Persistent HPV infection is an important risk factor for OPSCC [7,8]. Other factors including sex hormones, host immune background, oral sex practices, levels of infection, and tobacco smoking have been proposed as cofactors [[9], [10], [11]]. Yet, the mechanisms that explain sex differences, lesion location, and how HPV infection alone and in combination with cofactors contribute to OPSCC remain undefined [[12], [13], [14]]. The absence of early diagnostics and safe and effective treatments highlights the need to better understand HPV pathogenesis.

Marijuana (cannabis) usage has been linked to HPV OPSCC in patients [[15], [16], [17]]. Chronic marijuana use can have deleterious effects on several aspects of health [18,19]. At this time, with 1 in 16 youth in the U.S. report daily cannabis use, it remains the most widely used illicit drug [20]. As a consequence, cannabis use disorder (CUD) is an increasing problem among US adolescents [[21], [22], [23]]. Globally, marijuana is the most widely used illicit drug of abuse, with an estimated 2.6–5.0 % of people reporting marijuana use at least once in the previous year [24]. Of the more than 60 phytocannabinoids identified in marijuana samples, the two most abundant psychoactive ingredients include: Δ^9^-tetrahydocannabinol (THC) and cannabidiol (CBD) [25,26]. According to data from the National Institute on Drug Abuse (NIDA) data, the THC concentration in marijuana products has increased over time (up to 30 %), while the CBD content has typically remained low. The half-life of CBD and THC is reported to be between 2 and 7 days following chronic administration, and the total elimination of a single dose from one's urine can take up to 30 days, depending on the frequency of use and other factors [24,[27], [28], [29], [30]]. Currently, recreational cannabis use is legal for adults in 21 states, while medical use is legal in most other states to varying degrees. Oral HPV infection is detected six times more often in males than in females, and marijuana use is more prevalent among men [5]. However, it remains unclear whether marijuana contributes to the rising HPV-OPSCC epidemic in the United States [31]. Our previous study already demonstrated that smoke carcinogens such as Dibenzo [def,p]chrysene can accelerate squamous cell carcinoma in orally infected mice [32]. We anticipate an increase in marijuana use that could have an unprecedented impact on the rising incidence of HPV-associated OPSCC if the effects of marijuana on HPV-associated infections and cancers are not properly addressed. This study aims to investigate the role of the two major psychoactive ingredients of marijuana, THC and CBD, in HPV infection using a mouse model of papillomavirus (MmuPV1) that recapitulates HPV-associated oral infection and SCC [[32], [33], [34], [35], [36], [37], [38], [39], [40], [41], [42], [43], [44], [45]].

Cannabinoids are known to be lipid modulators that can act through two endocannabinoid receptors (CB1 and CB2) in both tumor-associated and non-tumor macrophages, dendritic cells (DCs), and leukocytes [46,47]. Cannabinoids affect the functioning of immune cells by suppressing phagocytosis, altering the expression of many cytokines, and impairing immune responses to influenza infections [[46], [47], [48], [49], [50]]. The MmuPV1 infects both immunocompetent and immunocompromised mice, targeting one of the two HPV-preferred sites in the human mouth for OPSCC [2], the base of the tongue, especially the circumvallate papilla (CVP) and adjacent Von Ebner's (VE) glands [34,35]. While infections are cleared in most immunocompetent mice [33], persistent infections and SCC can develop in immunocompromised mice at other sites of the oral mucosae and larynx, especially with co-risk factors such as tobacco carcinogens [32,[51], [52], [53]]. Therefore, the MmuPV1 model is the most suitable preclinical model to test our hypothesis that THC and CBD promote viral infection and suppress host immune responses. Our aim is to determine whether moderate use (3 times/week) at a modest dose (3 mg/kg) [25,28] of THC and CBD delivered intraperitoneally promotes papillomavirus infection and alters local host innate immune responses in athymic nude mice that show viral persistence in the oral cavity [34,35]. The modest dose in this pilot study is intended to mimic recreational or medical use in humans for treating chronic pain (10–25 mg/day/person) [54,55]. To investigate the systematic impact of THC and/or CBD on viral persistence, we infected athymic nude mice using a standard viral dose (1 × 10^9^ viral genome equivalents) in the current study. As these mice are deficient in T cell-mediated immune response, we focused on the impact of THC and CBD on some key innate immune cell populations, including dermal dendritic cells (CD205^+^CD11c^+^), granulocytes (Ly6G^+^), and macrophages (F4-80^+^), as demonstrated in our previous study [33]. All infected mice were followed up to 20 weeks post-infection with assessments of THC and CBD levels in the circulatory system, weight changes, and viral replication/persistence using established assays [54,56,57].

## 2 Materials and methods

### 2.1 Viral infection, oral swab, and oral tissue collection

All animal studies were reviewed and approved by the Institutional Animal Care and Use Committee of The Pennsylvania State University College of Medicine and followed NIH guidelines for care and use of animals in research. All mice were housed in the Pennsylvania State University College of Medicine, Association for Assessment and Accreditation of Laboratory Animal Care-approved, Animal Care Program. Both male and female athymic HSD: Nu nude mice were purchased from Envigo [58]. MmuPV1 infection of mouse oral tissues was achieved using previously described procedures [35]. In brief, 4–6-week-old mice were used for the current study. On day 0, all mice were anesthetized with ketamine (100 mg/kg)/xylazine (10 mg/kg), and the base of the tongue epithelium was gently wounded using microneedles [58]. All animals were allowed to recover overnight. The following day, each animal was again anesthetized, and virus inoculum (10 μl viral stock solution in saline, 1 × 10^9^ viral DNA in total) was placed onto the pre-wounded sites following additional gentle wounding with microneedles [33]. Oral swab collection started at week 4 post-infection by gently restraining mice and using a small saline-dipped plastic brush to swab the oral cavity, swirling it several times before placing it in a collection tube for DNA extraction and viral DNA quantification via qPCR [59]. Oral tissues were collected from mice at week 20 post viral infection, a time point that no squamous cell carcinoma was observed in MmuPV1 infected animals [32].

### 2.2 CBD and THC intraperitoneal delivery

Delta-9-tetrahydrocannabinol (THC) and cannabidiol (CBD) were obtained from the National Institute on Drug Abuse Drug Supply (Bethesda, MD). For all experiments, Δ^9^-THC and/or CBD were dissolved in 0.9 % saline, 5 % Cremaphor EL, and 5 % ethanol (18:1:1 v/v/v) and administered intraperitoneally (ip) [60]. Both purified CBD and THC (20 mg/ml stock) from the NIDA drug supply were dissolved to a concentration of 6 mg/ml before intraperitoneal (ip) delivery. All mice were treated with either THC (3 mg/kg), CBD (3 mg/kg), or CBD (3 mg/kg) plus THC (3 mg/kg) three days a week for the first four weeks and infected with MmuPV1 during week four. All mice in the vehicle control group also received the same volume of the diluent used for making up the working solution of other compounds according to body weight. Treatments were continued two times a week up to 20 weeks when the experiments were terminated.

### 2.3 CBD and THC in serum samples

The internal standard THC-d3 and CBD-d3 (100 mg/mL in methanol) were purchased from Cerilliant (a Sigma-Aldrich company, Round Rock, TX). Formic acid was purchased from J. T. Baker (New Jersey, USA). Optima LC-MS grade water, acetonitrile, methanol, and other chemicals were purchased from Fisher Scientific (New Jersey, USA). CBD and THC standard stock solutions were prepared in methanol. The standard working solutions were prepared by mixing THC and CBD from the standard stock solution in acetonitrile, followed by serial dilution, resulting in concentrations from 0.25 to 10,000 ng/mL. The internal standards working solutions were prepared by diluting the stock solution (100 mg/mL) using acetonitrile to make the concentration 100 ng/mL.

Standard curves were constructed by plotting the ratio of the peak area of the analyte to the peak area of the corresponding internal standard versus analyte concentration. The standard working solution of 4 mL and internal standard of 4 mL were spiked into 10 mL control plasma, and after vortexing, 22 mL acetonitrile/H2O/formic acid (90/10/0.1) was added to extract the analytes from plasma. Following vortexing and subsequent centrifugation at 8765 g for 10 min at 4 °C to precipitate proteins, the supernatant was taken and loaded onto the HPLC-MS-MS system, with final concentrations of 0.025 ng/mL to 1000 ng/mL for CBD and THC.

Treated plasma was processed the same way as the standards: after spiking 4 mL internal standards into 10 mL plasma, samples were vortexed, and 26 mL acetonitrile/H2O/formic acid (90/10/0.1) was added for extraction. The calculated concentrations from the standard curves were adjusted 4 times to reflect the in vivo levels of CBD and THC in plasma.

THC and CBD in plasma were analyzed using a Sciex QTRAP 6500+ mass spectrometer coupled with a Sciex EXion HPLC separation system. A 1.7 μm Acquity UPLC BEH C18 analytical column (2.1 × 100 mm, Waters, Ireland) was used to separate CBD and other isomers as well as impurities. The gradient elution was conducted using a flow rate of 0.4 mL/min with the following conditions: initial at 70 % mobile phase B (acetonitrile) and 30 % mobile phase A (0.1 % formic acid in water), followed by a linear gradient to 90 % mobile phase B in 1 min, and keeping the 90 % mobile phase B for 3 min to flush the column before returning to initial conditions to equilibrate the column.

The Sciex QTRAP 6500+ mass spectrometer was equipped with an electrospray ionization probe operated in positive ion mode. The mass spectrometry parameters for THC and CBD were as follows: declustering potential (DP) was 70 V; entrance potential (EP) was 10 V; collision energy (CE) was 33 V; and collision cell exit potential (CXP) was 12 V. The curtain gas (CUR) was 35 L/h, and the collision gas (CAD) was set to medium. The ionspray voltage was 5500 V, the temperature was 550 °C, gas 1 was 15 L/h, and gas 2 was 15 L/h.

The multiple reaction monitoring mode (MRM) was used to analyze and quantify CBD and THC as well as CBD-d3 and THC-d3, with the transitions of *m*/*z* 315 > 193 for CBD and THC, and 318 > 196 for CBD-d3 and THC-d3. All peaks were integrated and quantified using Sciex OS 3.0 software.

### 2.4 Viral load assessments

Viral titers in the oral swab samples were determined as previously described [33]. The swabs containing oral exfoliated cells were placed into saline in collection vials and total DNA was extracted using standard methods. Quantitative real-time PCR (qPCR) was performed using primers and probe targeting the E2 region (150 bp amplimer). Viral DNA genomes were amplified and quantitated using a standard curve of amplification from purified plasmid DNA containing a single viral genome [33]. The mean and standard errors (SEM) of the number of viral genomes per sample was plotted for each group.

Viral transcripts in the infected tissues were determined as previously described [59]. Total RNA was extracted from these tissues and reverse transcribed into cDNA for qPCR analysis. The copy numbers were based on 200 ng RNA from each sample (Viral RNA E1^E4 transcripts were quantified using primers 5′-TAGCTTTGTCTGCCCGCACT-3′ and 5′-GTCAGTGGTGTCGGTGGGAA-3′ and probe 5′FAM-CGGCCCGAAGACAACACCGCCACG-3′TAMRA. In brief, 200 ng of RNA was reverse transcribed using the RevertAid First Strand cDNA synthesis kit (Thermo-Fisher) and 2 μl cDNA was used in the qPCR analysis. 500 nM of each primer and 250 nM probe was used with the Brilliant III qPCR kit (Agilent) with the following qPCR conditions- 95 °C for 3 min, then 40 cycles at 95 °C for 5 s and 60 °C for 10 s on an Agilent AriaMx qPCR machine (Agilent) [33].

### 2.5 Cell disassociation for flow cytometry

All infected mice were sacrificed at week 18 post viral infection. The spleen and tongue were disassociated either by grinding or by collagenase disassociation medium (Sigma), respectively. Briefly, the tongue tissues were minced with a 21G scalpel blade and suspended in the collagenase solution (4 % BSA, 4 mg/ml collagenase type I, and 2 mg/ml collagenase type IV in DMEM) at 37 °C for 1.5 h with vortexing every 30 min [33]. The single cell suspension was harvested and stored in liquid nitrogen before flow cytometry analysis.

### 2.6 Flow cytometry

Antibodies targeting a panel of immune cell markers including CD45 (BV510), CD11b (M1/70, BV421), CD11c (N418, BV785), Ly6C (HK1.4, APC-CY7), Ly6G (1A8, PE-CY7), CD205 (NLDC-145, APC), CD207 (4C7, PE), F4/80 (BM8, BV711) or CD68 (FA-11, BV711), Siglec F(S17007L, PE-CF594), and Siglec H (551, PerCP/Cyanine5.5) were obtained from Biolegend and used as recommended by the manufacturer [33]. Cells were also labeled with a fixable viability dye (Biolegend) to gate the viable cells. Suspended cells from spleen and tongue samples were labeled and analyzed by multi-color flow cytometry using an LSR Fortessa in the Flow Cytometry Core of the Penn State College of Medicine. Cell populations were gated on live CD45 positive cells prior to subset discrimination using FlowJo 10.1r8 software [33].

For multi-color flow cytometry, single viable leukocytes (CD45^+^) from either spleens or tongues were gated for subset discrimination as described previously [33]. Total dendritic cells (CD11c^+^F4-80^-^) by exclusion of macrophages (F4-80^+^) were further analyzed to determine two dendritic cell populations including lymphoid dendritic cells (CD11b^low^CD11C^+^, cDC1) and myeloid dendritic cells (CD11b^high^ CD11C^+^, cDC2). Dermal DCs (CD205+) of total DCs and plasmacytoid DCs (CD11b^low^ SiglecH^+^) from cDC1 were further determined. Macrophages (CD68^+^) and granulocytes (Ly6G^+^) were further analyzed based on CD11b ^high^ cell population. The numbers indicate the percentages of each cell population in the parent viable single CD45^+^ cells. Cell populations were gated on live CD45 positive cells prior to subset discrimination using FlowJo 10.1r8 software [33].

### 2.7 Statistical analyses

For outcome variables measured repeatedly over the course of treatment (such as body weight and viral loading from oral swab), linear mixed-effect models were used to evaluate the overall change of outcome over time. In general, the analyses were stratified by sex. Within each sex, the comparisons among treatment groups at each time point were made using One-way ANOVA models. For other outcome variables that do not involve repeated measures, their values among different treatment groups and sex were visualized using mean ± standard error of mean (SEM) plots. Similarly, ANOVA models were used among treatment groups within each sex. All analyses were performed using statistical software SAS version 9.4 (SAS Institute Inc., Cary, NC, USA). All tests were two-sided, and the statistical significance level used was 0.05. Due to the exploratory nature of this study, the significance level was not adjusted for multiple testing. The graphs were generated in either Sigmaplot 11 (Systat Software Inc., San Jose, CA, USA) or GraphPad Prism 10 (GraphPad Software, Boston, MA, USA).

## 3 Results

### 3.1 CBD and THC were detected in the serum samples of treated mice

**Fig. 1:**
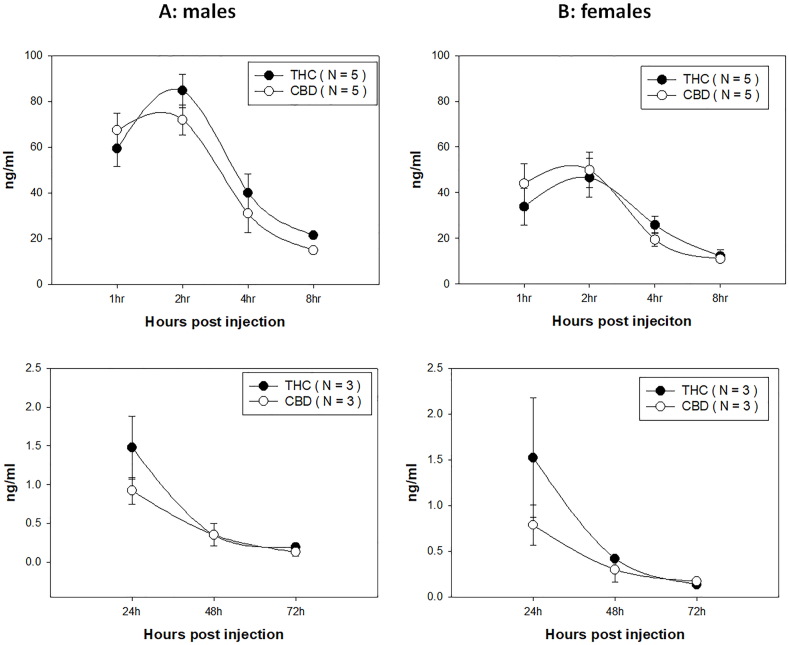
THC and CBD were detected in the serum samples of both male (A) and female (B) mice up to 72 h post-intraperitoneal delivery. The levels of THC and CBD with decreased in both sexes after 2 h. A significant difference was found between male and female mice that received both THC and CBD at 1 and 2 h, partially due to the concentration of CBD and THC being administered according to body weight (the top panel). However, comparable levels of THC and CBD were detected after 24 h post-injection (the bottom panel).

Intraperitoneal (ip) administration of CBD and THC can be absorbed and transmitted to the blood and detected by HPLC. We monitored THC and CBD concentrations in the mouse blood after ip administration. We first established an extremely sensitive method using our advanced HPLC analysis in the Penn State COM core facility. The lowest concentration we could measure was 0.1 ng/ml (Fig. 1). To track CBD and THC levels in ip-injected mice, we collected blood samples at 1, 2, 4, 8, 24, 48, and 72 h post-administration and checked their levels. We detected CBD and THC in our tested mice around 1-h post-injection, suggesting ip injections are effective for delivering both THC and CBD into the circulatory system quickly. As shown in Fig. 1, we can detect THC and CBD in the blood of both male (Fig. 1A) and female (Fig. 1B) mice. A similar trend of decreasing detection of both THC and CBD was found in both sexes. Not surprisingly, significantly higher levels of THC and CBD were found in males up to 8 h post administration compared to those in females partially due to the concentration of CBD and THC being administered according to body weight. Extremely low but detectable levels of THC and CBD were found up to 72 h post-delivery with no sex differences detected at that time point. The levels of both THC and CBD were below the legal limits reported in current human standards (2 ng/ml) after 24 h [29,61].

### 3.2 Body weight was not affected by THC, CBD, or THC + CBD administration

**Fig. 2:**
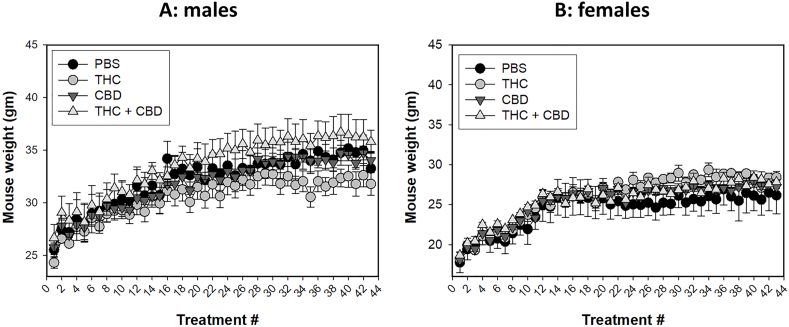
A similar trend in weight gain was observed over the course of treatment. No difference in body weight was found in male (A) or female (B) mice among the THC, CBD, or THC + CBD treated groups compared to the vehicle control group.

Marijuana has been linked with weight changes, either gain or loss, in users [[62], [63], [64], [65], [66], [67]]. The potential effects of ip delivery of THC and CBD on weight gain remain unclear. We monitored the weight of the mice after each treatment. Four groups were included in this study: vehicle control, THC, CBD and THC + CBD. As shown in Fig. 2, males are heavier than females. However, no significant weight differences were observed within male (Fig. 2A) or female (Fig. 2B) mice across the treatment groups, suggesting that the current treatment did not impact weight in either sex.

### 3.3 Increased viral DNA copies were detected in infected mice

**Fig. 3:**
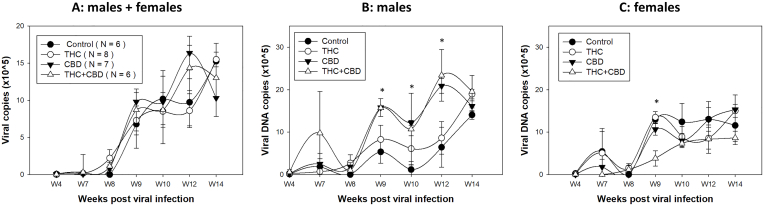
Orally infected mice of all groups showed increased viral copies over the time course of infection in the oral swabs collected longitudinally (A). Increased viral DNA levels were found in CBD and T + C treated males at week 9 (p = 0.0169), 10 (p = 0.0261) and 12 (p < 0.0001) compared to the control males (B). In contrast, T + C treated females showed significantly decreased viral DNA at week 9 (p = 0.0047) post viral infection (C).

Next, we wanted to determine whether ip delivery of a modest dose (3 mg/kg) of THC, CBD, or THC + CBD (T + C) with moderate use (3 times/week) promotes viral replication and persistence in athymic nude mice. Vehicle controls were included using the same volume according to body weight. Animal tongues of all groups (N = 3–4/group of male or female mice) were infected with MmuPV1 (1 × 10^9^ viral DNA in total) after four weeks of ip injection [33], followed by a treatment 2 times/week, until experimental termination at week 20. Due to the limited number of cells that could be collected from oral swabs for viral transcript quantification, we focused on measuring viral DNA by collecting oral swabs from four weeks post-infection up to 14 weeks post-infection. In agreement with a previous study [35], very low levels of viral DNA were found in both males and females before week 8 post-infection. The fluctuation in viral DNA detection also indicated some variations in collecting infected cells from the base of the tongue, an area that is also challenging to reach for HPV detection [68]. Overall when combined, slightly higher but not significantly higher levels of viral DNA were found in CBD and THC + CBD treated mice (Fig. 3A). However, when separated by sex, significantly higher levels of viral DNA were found in males of the CBD and THC + CBD groups at week 9 (p = 0.0169), 10 (p = 0.0261) and 12 (p < 0.0001) compared to the control males, but not at other time points (Fig. 3B). In contrast, females of the THC + CBD group showed decreased viral DNA at week 9 (p = 0.0047) but not at other time points (Fig. 3C). The detection of viral DNA increased in the oral swabs over the course of infection for all groups. This agrees with our previous observations, and shows that all mice were susceptible to MmuPV1 infection in the oral cavity [32,35]. These data suggest that CBD but not THC may play a role in promoting viral replication in the oral cavity of infected male mice.

### 3.4 Viral RNA was detected in the CVP region and secondary infection was detected in orally infected mice

**Fig. 4:**
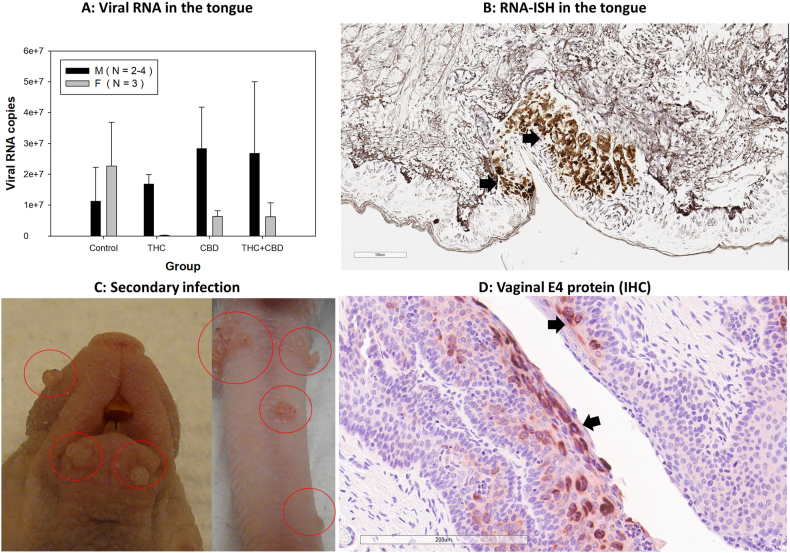
Viral RNA Detection in Infected Tongues and Secondary Infection at Skin and Mucosal Sites. Slightly higher levels (but not statistically significant) of viral RNA was observed in all treated male groups compared to the controls. Intriguingly, the opposite was found in females (A). Viral RNA was detected in the infected cells at the base of the tongue (B, indicated in brown, arrows). Secondary infections were also detected in several cutaneous tissues with visible lesions (C, circled). Viral signals were found in the vaginal tissues by immunohistochemistry (IHC) for MmuPV1 E4 (D, indicated in red, arrows), consistent with our previous report.

To further determine changes in the infected tissues, all infected tongues were harvested for additional assessment of viral RNA. Our previous report suggested slightly higher levels of viral transcripts in the infected tongues of immuncompetent C57BL/6 (B6) male mice compared to female B6 mice [33]. There was no statistically significant difference in viral RNA transcripts in THC, CBD and T + C compared to those in the control group (Fig. 4A) although males were marginally higher in all treatment groups compared to the control. Consistent with previous studies, viral RNA was detected at the base of the tongue, particularly in the CVP region (Fig. 4B) [[33], [34], [35],^43^,^59^]. Interestingly, but not surprisingly, secondary infections were also observed at several cutaneous sites, such as the tail and muzzle, where visible lesions were present (Fig. 4C) [43]. Additionally, secondary infections in the anogenital tissues were evident, with representative samples from vaginal tissues testing positive for viral E4 via IHC (Fig. 4D). These findings suggest that the orally infected mice transmitted the virus to other sites, likely through self-grooming or contact with cage mates [43,69].

### 3.5 A sex difference in granulocytes, dendritic cells but macrophages was found in the infected tongues of CBD treated mice

**Fig. 5:**
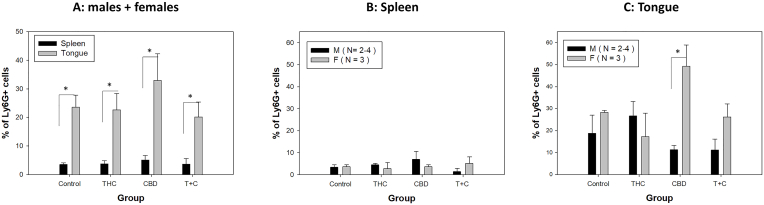
Ly6G + Granulocytes Analysis in Spleen and Tongue of Infected Mice. Overall increased Ly6G + granulocytes were observed in the infected tongues compared to the spleens of all groups (A). No differences were found among all tested groups or between sexes in spleens (B). Significantly higher levels of Ly6G + granulocytes were found in CBD-treated females compared to corresponding males (C, ∗p = 0.0017), but not in other groups.

Our previous study showed a sex difference in the local myeloid infiltration in immunocompetent B6 mice infected orally with MmuPV1 [33]. We therefore examined whether THC and/or CBD influenced the local myeloid cells in the infected tongues of our tested mice. The athymic nude mice have no T cell-mediated immune responses and therefore no T cell- or B cell-mediated immune responses are generated [70]. Using the same panel of antibodies, we focused on dermal dendritic cells (CD205+), granulocytes (Ly6G+), and macrophages (F4-80+). In brief, single viable CD45^+^ cells were analyzed based on different CD markers to determine various immune cell populations in the spleens and tongues of infected mice. Different myeloid cell populations, including dermal dendritic cells (CD205+), granulocytes (Ly6G+), and macrophages (F4-80+), were analyzed as we described previously [33]. Overall, significantly higher levels of granulocytes (Ly6G+) were found in tongues compared to those in spleens in all tested groups (Fig. 5A). There was no significant difference in granulocytes (Ly6G+) in the spleens of all groups (Fig. 5B). However, significantly higher levels of granulocytes (Ly6G+) were recruited to the female tongues of CBD treated mice compared to the corresponding males (p = 0.0017) (Fig. 5C). No difference was found in granulocytes (Ly6G+) in the infected tongues of the males among all groups (Fig. 5C).

**Fig. 6:**
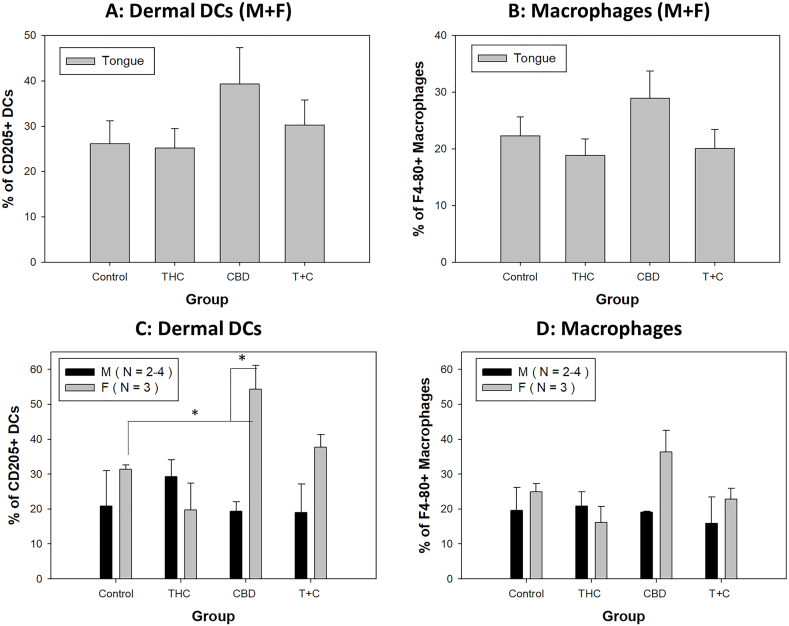
No significant difference among different groups was found in both dermal DCs (A) or macrophages (B) in the infected tongues when combining both male and female together. Significantly higher numbers of dermal DCs were found in females of the CBD-treated group compared to corresponding males (C, p = 0.0173). Significantly higher levels of dermal DCs (CD205+) were observed in CBD-treated females compared to the control group (C, p = 0.019). D) No significant difference between the tested groups vs the control group although significantly higher levels of macrophages (F4-80+) were found in CBD-treated females compared to THC-treated females (p = 0.013). No significant differences were found in both dermal DCs (CD205+) and macrophages (F4-80+) among all males.

Two dendritic cell populations including lymphoid dendritic cells (CD11b^low^CD11C^+^, cDC1) and myeloid dendritic cells (CD11b^high^CD11C^+^, cDC2) were initially analyzed as previously described [33]. Dermal DCs (CD205+) were further determined with F4-80+ macrophages being subtracted. Comparable numbers of different DC subsets (of the parent CD45^+^ population) were found between females and males in spleens of the infected animals (data not shown). No significant differences in numbers of both dermal DCs (Fig. 6A) and macrophages (F4-80^+^) (Fig. 6B) were found among these groups when both males and females combined. Consistent with our previous findings in immunocompetent mice, including B6 mice, we also detected a sex difference in dermal DCs in the CBD treated group (Fig. 6C, p = 0.0173). No sex difference was found in other groups. Significantly higher levels of dermal DCs (CD205+) were found in the CBD treated females compared to the control (p = 0.019) (Fig. 6C). Even though significantly higher levels of macrophages (F4-80+) were found in CBD treated females compared to THC treated females (Fig. 6D, p = 0.013), no significance was found in dermal DCs (CD205+) and macrophages (F4-80+) among all males.

## 4 Discussion

This study is the first to demonstrate that intraperitoneal administration of CBD, one the two main components of cannabis (THC and CBD), can promote viral infection and alter innate immune responses in immunocompromised athymic nude mice [33]. We were able to detect THC and CBD in the serum of treated mice up to 72 h post-treatment, with levels relevant to detection in human marijuana users. We also detected a higher viral load in males of the CBD and THC + CBD treated groups compared to the vehicle control group in orally MmuPV1 infected mice at some time points post infection. Only marginally higher levels of viral RNA were detected in the infected tongues of treated males, however. Interestingly, a similar sex bias was observed in several myeloid cells recruited to the infected tongues, as we previously reported [33]. Furthermore, we observed secondary cutaneous and mucosal infections in these orally infected mice, as reported previously [43,69]. Given that we tested only moderate doses of THC and CBD, the impact on viral replication and persistence, as well as host innate immune responses, suggests a potential effect of cannabis on HPV-associated diseases and cancers in humans. However, further experiments are needed to determine the long-term effects.

Although previous work suggests that marijuana consumption can impact HPV oral infection [5], the role of marijuana use in the increased incidence of HPV-associated OPSCC remains elusive. Negative side effects associated with daily marijuana inhalation include changes to airway conductance [62,71], and increased squamous cell metaplasia when both tobacco and marijuana smoking occur simultaneously [[72], [73], [74]]. In addition to smoking, the use of THC and CBD oil are other consumption methods that are commonly utilized by marijuana users [75]. Better controlled studies are needed to determine how marijuana and its two major cannabinoid constituents (THC and CBD) impact oral HPV infection and HPV-associated OPSCC. This preliminary study is the first to use a mouse papillomavirus infection model to address this question.

Clinically, the half-life of CBD and THC was reported to be between 2 and 7 days after chronic oral administration in human studies [27]. We tested moderate use (3 times/week) with a modest dose (3 mg/kg) of THC and CBD that is relevant dose for human consumption based on allometric interspecies dose scaling [76,77]. This dose is relatively low compared to what has been commonly used in other animal studies (10–50 mg/kg) because we wanted to use a translationally relevant dose in our study [78]. Intriguingly, we were able to detect THC and CBD in the plasma up to 72 h post intraperitoneal delivery using a method that is extremely sensitive to detect exceptionally low levels of THC and CBD (0.1 ng/ml). Most studies on THC and CBD in preclinical models have focused on behavior and used intraperitoneal (i.p.) or subcutaneous (s.c.) injections [[79], [80], [81], [82], [83], [84]]. However, the pharmacokinetics of THC in plasma were monitored only some of these studies [81,85,86]. These studies monitored THC in the plasma for up to 26 h post administration, using measures of ng/ml [81] or pmol/ml [85,86]. In the current study we tracked THC in the plasma up to 72 h post ip injection. In contrast to the previous study using C57BL/6 mice and showed highest levels of THC in the plasma around 1 h post ip administration [85,86], both compounds peaked around 2 h but decreased to a legal human serum level around 24 h post-administration in the tested athymic mice in the current study. The levels of THC in the plasma between our study and the previous study are comparable. Interestingly, THC and CBD showed a similar trend of metabolism in the blood, although THC levels were higher compared to CBD, which is consistent with what has been shown in animal and human studies [60,87]. As we administered THC based on body weight, significantly higher levels of THC and CBD were detected in males, consistent with previous studies using ip delivery [85,86]. Chronic marijuana users have plasma THC concentrations ranging from 1.0 ng/ml to 11.0 ng/ml [29,61]. Our detected concentrations in THC-treated mice fall in this range, which mimics human conditions [29,88,89]. Therefore, we can use the mouse model to mimic different scenarios for recreational human marijuana use.

The impact of marijuana on body weight has been mixed [63,65,67]. Clinically, short-term marijuana smoking is linked with weight gain due to acute marijuana use being associated with hyperphagia [65]. A preclinical mouse study demonstrated that long-term stress and cannabis exposure significantly reduced body weight gain [63]. In the current study, we did not detect significant weight changes among all treated groups, suggesting that the dose and regimen used in our study did not affect weight.

We detected increased viral DNA copies in the oral swabs of both CBD and THC + CBD treated male mice compared to the control mice, suggesting that CBD may promote viral replication in the oral cavity of males but not females, as we detected an opposite result in the female CBD + THC treated group. Viral detection in the oral cavity was found to be delayed compared to the lower genital tract in the same infected mouse of this athymic nude mouse strain [35]. In the current study, we detected very low levels of viral DNA from all groups before week 8 post-infection, but with increased levels in CBD and THC + CBD but not THC treated males. The increased viral DNA copies at earlier time points post-infection suggest increased shedding of virus-infected cells and viral replication of these cells in the oral cavity. This standard dose (1 × 10^9^ viral genome equivalent) for oral infection is relatively high and may have masked the impact of THC and CBD on viral life cycle in the oral cavity, leading to only slightly higher levels of viral DNA/RNA in both test groups at later time points (20 weeks post-infection). A similar phenomenon was noticed in our published study, where a much lower dose of viral DNA could establish infection and induce squamous cell carcinoma at similar time points post-infection in the genital tract [90]. We postulate that a lower dose of virus infection might be able to distinguish this difference in future studies.

The immune system plays a crucial role in recognizing and eliminating HPV-infected cells [[91], [92], [93]]. HPV-associated OPSCC is more prevalent in immunosuppressed populations [3,92]. In the current study, we used an immunocompromised athymic nude mouse strain without adaptive immunity, which was proven to be susceptible to MmuPV1 infection with viral persistence in both cutaneous and mucosal tissues [34,35,39]. Because these mice are deficient in generating adaptive immunity, we investigated changes in several innate immunity-associated myeloid cells [39]. Interestingly, only CBD altered several myeloid immune cell infiltrations, including dermal dendritic cells (CD205+), granulocytes (Ly6G+), but not macrophages (F4-80+), at the infected tongues, with a sex difference observed. Sex differences in these tested immune cell populations were not observed in the THC + CBD group, although both groups showed increased viral replication in the oral swab. One explanation is that THC might counteract some impact of CBD on the local immune cell landscape through an unknown mechanism, which needs further investigation. THC and CBD have been reported to affect the functioning of immune cells by suppressing phagocytosis, altering the expression of many cytokines, and impairing immune responses to influenza infections [[46], [47], [48], [49], [50]]. Whether these myeloid cells provided a protective effect for the female tongues against MmuPV1 infection is unclear. The sex difference in recruiting different innate immune cells to the infected tissues may suggest that males and females use different strategies to respond to viral infections [33]. Historically, men have a higher incidence of HPV infection and associated oropharyngeal cancer than females. Men are also more likely to engage in risk behaviors, including drug abuse. Marijuana use is much higher in men than in women. Our observed increased viral load in males aligns with these reports. In our previous studies using immunocompetent mice, we also detected increased levels of some immune cells in females, leading us to postulate that these immune cells may provide a protective effect for females. Functional assays would be able to determine how these immune cells help in viral control in females. We did not detect more severe histology in THC- and CBD-treated animals compared to the control mice, as we observed in our previous study with a tobacco carcinogen [32]. A longer-term study is needed to understand the impact of THC and CBD on tumor progression by following disease progression from low to high-grade dysplasia in these chronic THC and CBD treated mice. How sex hormones play a role in this unexpected finding remains unclear and additional studies are needed to address this important question.

There are a few limitations to consider in the interpretation of our results. First, we detected variation and low levels of viral DNA in oral infections at earlier time points post-infection as reported in our previous studies [[33], [34], [35]]. Second, we used a small sample size for this pilot study. Due to individual differences, the findings in some experiments may be biased and impact our interpretation. Nonetheless, our conclusions are plausible, especially regarding the sex differences in several innate immune cells after CBD treatment. We also reported sex difference in some of these innate immune cells during viral clearance in immunocompetent mice [33]. Our findings further suggest that males and females may use different components of innate and adaptive immune responses to control oral papillomavirus infection.

Overall, our study demonstrated that the intraperitoneal administration of THC and CBD can be readily detected in serum at levels comparable to those found in human marijuana users. CBD administration showed increased viral detection in the oral swabs and infected tongues of athymic nude male mice. A longer-term study with increased frequency of administration is warranted to mimic the effects of chronic consumption on HPV infection and OPSCC in vulnerable immunocompromised populations who use these drugs. We also detected secondary infections in both cutaneous (tail and muzzle) and mucosal (vagina) tissues of these infected nude mice, further confirming our earlier observations of transmission from one site to another and potential blood transmission [34,35,43,69]. It is well known that marijuana users may engage in other risky behaviors, leading to a higher risk of exposure to HPV [77,94,95]. Further studies will determine whether long-term administration of THC and CBD will increase oropharyngeal squamous cell carcinomas in these mice, as we previously reported that a tobacco carcinogen promoted SCC [32]. It would also be interesting to decipher which cannabis receptor pathways (CB1 and/or CB2 receptors) are involved using selective antagonists for CB1 (SR141716; Rimonabant) and CB2 (SR144528), as well as commercially available CB1 and CB2 knock-out mice in future studies [96]. These studies will help define their roles in HPV-associated infections and cancers in different cannabis users. Remaining questions include whether medical cannabis prescribed to chemotherapy patients with cancer promotes HPV infection and associated diseases, as this group of people is more likely to be immunocompromised to some extent due to treatment. Furthermore, are adolescents using illicit drugs more likely to engage in other risky behaviors, such as tobacco smoking, drinking, and unsafe sex? Our previous study already demonstrated that smoke carcinogens Dibenzo [def,p]chrysene can accelerate squamous cell carcinoma in orally infected athymic nude mice [32]. More studies are needed to address these intriguing questions to test the combination of these co-factors. The sex differences observed in CBD-treated groups may lead to studies dissecting the role of sex hormones and receptors in HPV-associated infections and persistence.

## CRediT authorship contribution statement

**Sarah A. Brendle:** Writing – review & editing, Validation, Methodology, Investigation, Formal analysis, Data curation. **Jingwei Li:** Writing – review & editing, Visualization, Validation, Methodology, Investigation, Formal analysis, Data curation. **Dongxiao Sun:** Writing – review & editing, Validation, Software, Methodology, Investigation, Formal analysis, Data curation. **Junjia Zhu:** Writing – review & editing, Visualization, Validation, Software, Formal analysis, Conceptualization. **Angela N. Henderson-Redmond:** Writing – review & editing, Validation, Investigation, Conceptualization. **Daniel J. Morgan:** Writing – review & editing, Validation, Resources, Investigation, Conceptualization. **Karla K. Balogh:** Writing – review & editing, Visualization, Validation, Methodology, Investigation, Formal analysis, Data curation. **Danielle Covington:** Writing – review & editing, Validation, Methodology, Investigation, Data curation. **Debra A. Shearer:** Writing – review & editing, Visualization, Validation, Methodology, Investigation, Formal analysis, Data curation. **Jiafen Hu:** Writing – review & editing, Writing – original draft, Visualization, Validation, Supervision, Resources, Project administration, Methodology, Investigation, Funding acquisition, Formal analysis, Data curation, Conceptualization.

## Ethical approval

All animal studies were reviewed and approved by the Institutional Animal Care and Use Committee of The Pennsylvania State University College of Medicine (PROTO201800577, approved 2/18/2019) and followed NIH guidelines for care and use of animals in this research.

## Data availability statement

The data supporting the finding of this study are available from the corresponding author upon request.

## Conflicts of interest

The authors declare no conflict of interest.

## Funding

This work was supported by the following: 10.13039/100000072National Institute of Dental and Craniofacial Research grant no. R21DE028650 (J.H.), 10.13039/100011597Penn State Cancer Institute Program Project Development Award Sponsored by Highmark Community 10.13039/100018696Health Reinvestment Fund; The 10.13039/100020950Department of Pathology and Laboratory Medicine, the Jake Gittlen Memorial Golf Tournament.

## Declaration of competing interest

None.

## Data Availability

Data will be made available on request.
